# Granulation of Lithium-Ion Sieves Using Biopolymers: A Review

**DOI:** 10.3390/polym16111520

**Published:** 2024-05-28

**Authors:** Inimfon A. Udoetok, Abdalla H. Karoyo, Emmanuel E. Ubuo, Edidiong D. Asuquo

**Affiliations:** 1 Department of Chemistry and Biochemistry, University of Regina, Regina, SK S4S 0A2, Canada; 2 Lithium Research Centre, Arizona Lithium, 615 W Elliot Rd, Tempe, AZ 85284, USA; 3 Research and Development, Nortek Data Center Cooling, 1502D Quebec Ave, Saskatoon, SK S7K 1V7, Canada; 4 Department of Chemistry, Akwa Ibom State University, Mkpat Enin 532111, Nigeria; eubuo@yahoo.co.uk; 5 Department of Chemical Engineering, The University of Manchester, Manchester M13 9PL, UK; edidiong.asuquo@manchester.ac.uk

**Keywords:** granulation, biopolymer, composite, lithium-ion sieves (LISs), adsorption capacity, crosslinking, mechanical strength

## Abstract

The high demand for lithium (Li) relates to clean, renewable storage devices and the advent of electric vehicles (EVs). The extraction of Li ions from aqueous media calls for efficient adsorbent materials with various characteristics, such as good adsorption capacity, good selectivity, easy isolation of the Li-loaded adsorbents, and good recovery of the adsorbed Li ions. The widespread use of metal-based adsorbent materials for Li ions extraction relates to various factors: (i) the ease of preparation via inexpensive and facile templation techniques, (ii) excellent selectivity for Li ions in a matrix, (iii) high recovery of the adsorbed ions, and (iv) good cycling performance of the adsorbents. However, the use of nano-sized metal-based Lithium-ion sieves (LISs) is limited due to challenges associated with isolating the loaded adsorbent material from the aqueous media. The adsorbent granulation process employing various binding agents (e.g., biopolymers, synthetic polymers, and inorganic materials) affords composite functional particles with modified morphological and surface properties that support easy isolation from the aqueous phase upon adsorption of Li ions. Biomaterials (e.g., chitosan, cellulose, alginate, and agar) are of particular interest because their structural diversity renders them amenable to coordination interactions with metal-based LISs to form three-dimensional bio-composite materials. The current review highlights recent progress in the use of biopolymer binding agents for the granulation of metal-based LISs, along with various crosslinking strategies employed to improve the mechanical stability of the granules. The study reviews the effects of granulation and crosslinking on adsorption capacity, selectivity, isolation, recovery, cycling performance, and the stability of the LISs. Adsorbent granulation using biopolymer binders has been reported to modify the uptake properties of the resulting composite materials to varying degrees in accordance with the surface and textural properties of the binding agent. The review further highlights the importance of granulation and crosslinking for improving the extraction process of Li ions from aqueous media. This review contributes to manifold areas related to industrial application of LISs, as follows: (1) to highlight recent progress in the granulation and crosslinking of metal-based adsorbents for Li ions recovery, (2) to highlight the advantages, challenges, and knowledge gaps of using biopolymer-based binders for granulation of LISs, and finally, (3) to catalyze further research interest into the use of biopolymer binders and various crosslinking strategies to engineer functional composite materials for application in Li extraction industry. Properly engineered extractants for Li ions are expected to offer various cost benefits in terms of capital expenditure, percent Li recovery, and reduced environmental footprint.

## 1. Introduction

**Figure 1:**
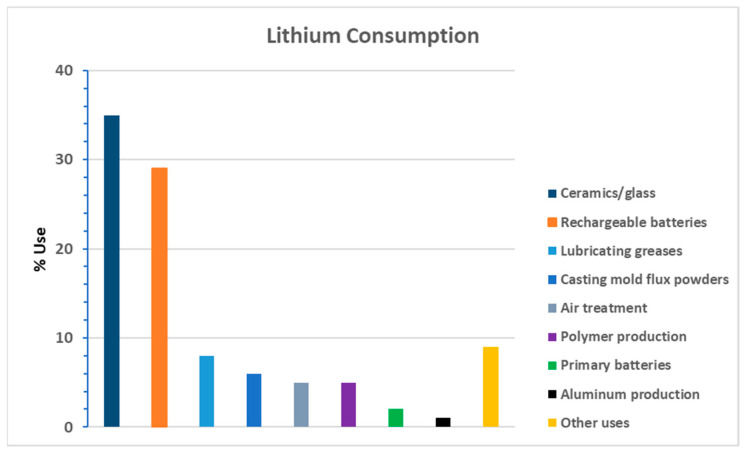
Histogram showing end uses of lithium as a percentage of world consumption. Adapted from Weng at al. [3].

The transition to clean renewable energy storage devices, along with the growing popularity of EVs, has led to massive interest in Li production. Li has other attractive applications in various manufacturing sectors, including non-rechargeable batteries, ceramics, glasses, lubricating greases, electrocatalysis, and medicine, among other uses (Figure 1). The utility of Li in the clean renewable energy sector (e.g., rechargeable batteries) relates to its electrochemical activity and high specific heat capacity [1]. The excellent charge-holding capacity of Li and the efficient delivery of the stored power make this element attractive from an application and commercial perspective. According to the United States Geological Survey (USGS) [2], natural Li exists in various ores, including continental brines, claystone, geothermal brines, hectorite, oilfield brines, and pegmatites.

**Figure 2:**
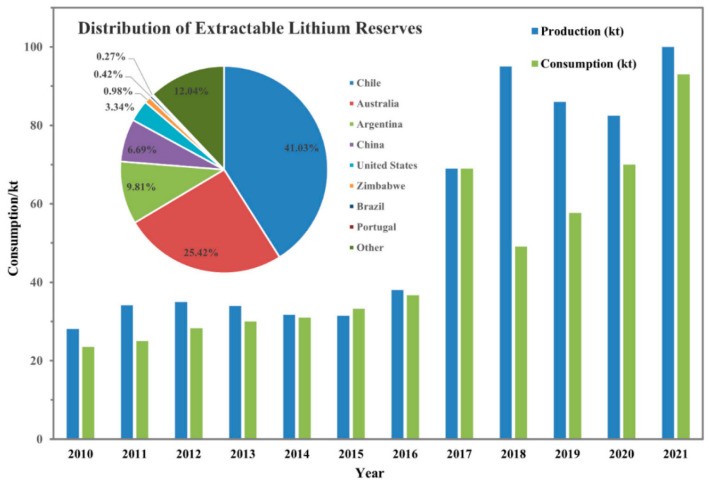
Global consumption of Li content from 2010 to 2020 and distribution of extractable Li reserves with existing technologies (reproduced from [6] with copyright permission).

The global distribution of extractable Li reserves varies, as summarized in the pie chart in Figure 2. The bar graph in Figure 2 shows the global consumption of Li for the 2010–2020 period, whereas its production is based on existing technologies. It is estimated that the annual demand for elemental Li will increase from 150,000–190,000 tons in 2025 to about 400,000–700,000 tons in 2100 [4,5]. This high demand, along with the massive natural reserves of Li, suggests that there is a dire need for the development of facile and feasible technologies for its recovery.

Several technologies for extracting Li ions from aqueous resources exist, such as solar evaporation, chemical precipitation [7,8], solvent extraction [6,9], adsorption [6,10], electrochemical methods [5,6,11], membrane-based technologies [6,12,13,14,15], and reaction-coupled separation methods [16,17,18]. Conventional evaporation-based methods are limited because they are not suitable for processing brine with high magnesium-to-lithium (Mg/Li) ratios. Additionally, such methods are time-consuming and involve complex processes to remove the residual Mg ions [6,19,20]. Similarly, solvent extraction technology is undesirable due to the high cost and toxicity of organic solvents used, along with the significant loss of extractant in the aqueous phase [21,22]. Furthermore, a poor understanding of the extraction mechanisms suggests that the use of this technique may be subjected to unfavorable outcomes. Adsorption-based technologies are the most feasible and scalable processes due to various advantages: they are simple, have a low carbon footprint, and the adsorbents can recover Li ions at high capacity while rejecting undesired contaminants [6,23,24,25,26], proving to be the most cost-effective and efficient method of Li extraction. Adsorption technology is important for the Li-ion extraction industry because it enhances the high recovery of Li ions, thus improving the economics of the extraction process at a commercial scale. Various organic (e.g., crown ethers and polymer ion-exchange resins) and inorganic (e.g., Al, Mn, and Ti-based) materials have been used as adsorbents for Li ions extraction [6,23,25,27,28]. Metal-based adsorbent materials are the most popular due to their relatively lower cost of production, facile synthetic processes, high Li ions recovery, excellent contaminant rejection, and good cycling performance. However, the nano-/micron-sized metal-based adsorbents suffer from poor mechanical strength and challenges associated with isolating the Li-ion-loaded adsorbents from the aqueous phase [24].

Granulation of metal-based Lithium-ion sieves (LISs) using biopolymer binding agents has been reported [3,6,24,29] to produce composite materials with engineered properties such as mechanical strength, porosity, bulk density, and particle size distribution for tailored applications [26,30]. The use of binding agents (e.g., biopolymers, synthetic organic polymers, and inorganic materials) afford LISs that can be easily isolated from the aqueous media, thus enhancing their utility in large-scale industrial applications. It is worth noting that the granulation of metal-based LISs may be accompanied by reduced uptake properties due to the attenuation of accessible binding sites for Li ions adsorption. However, a properly engineered granulation process can ensure that the properties of the LISs granules are optimized in terms of both phase separation and adsorption capacity performances. Moreover, various crosslinking strategies of the LISs prior to and post-granulation have been reported to further enhance the mechanical stability of the LISs granules. The current review highlights recent progress in the use of biopolymer binding agents for the granulation of metal-based adsorbent materials for Li-ion extraction. The study further reviews the effects of various biopolymer binding agents on the properties of the granulated composite materials, such as adsorption capacity, selectivity, isolation, recovery, and cycling performance. The adsorbent granulation process using biopolymer binders has been reported to modify the uptake properties of the composite materials to varying degrees in accordance with the surface and textural properties of the binding agent. The current study also highlights the importance of granulation technology and various crosslinking strategies for improving the Li ions extraction performance and mechanical properties of the LIS granules, along with their amenability for column operations in large-scale industrial applications.

## 2. Preparation of Lithium-Ion Sieves: An Overview

LISs represent simple inorganic composite materials that comprise different metallic oxides and/or carbonates. They are generally categorized according to their structural composition, where LMOs (lithium manganese oxides) and LTOs (lithium titanium oxides) are reported as the major classes. The nature and type of the precursors determine the affinity of the LISs for Li ions. Furthermore, the relative ratio of Li and the precursor ion (e.g., Mn or Ti) affects the performance and regeneration ability of the LISs due to changes in crystallinity. LMO-type LISs, such as λ-MnO_2_, MnO_2_·0.3H_2_O, and MnO_2_·0.5H_2_O, have been synthesized from LiMn_2_O_4_, Li_1.33_Mn_1.67_O_4_ (Li_4_Mn_5_O_12_), and Li_1.6_Mn_1.6_O_4_ (Li_2_Mn_2_O_5_) precursors, with high Li adsorption capacities. Similarly, LTO-type LISs, H_4_Ti_5_O_12_, and H_2_TiO_3_, derived from Li_4_Ti_5_O_12_ and Li_2_TiO_3_ precursors, have been reported to provide high recovery of Li from aqueous media. Other more complex inorganic precursors have been reported, such as LiFeMnO_4_, LiMg_0.5_Mn_1.5_O_4_, LiSbO_3_, LiAlMnO_4_, and LiNbO_3_ [31,32]. The reader is referred to an amplitude of references that have reported on the various types of LISs and their preparation strategies [3,6]. The focus of this section is to provide a general strategy for the preparation of LISs.

**Scheme 1:**
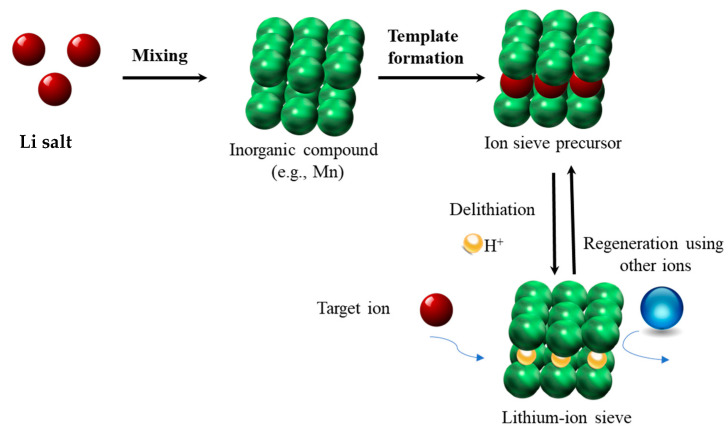
Schematic representation of the preparation process for lithium ion-sieves.

The preparation of LMO-type LISs will be used as a reference and consists of two steps: (1) preparation of precursors and (2) acid treatment to intercalate the Li ions. The general strategy for the preparation of LISs is depicted in Scheme 1 and is herein described. Several synthetic methods for precursor preparation through Mn and Li templation have been developed. These methods are categorized as solid-phase and soft-chemical methods. In the solid-phase method, Li and Mn salts are mixed according to a certain stoichiometric ratio, followed by calcination at an appropriate temperature to obtain the corresponding products [3,33,34,35,36]. Solid-phase methods are simple and easy to apply; however, they suffer from uneven mixing and inadequate reaction of raw materials. By contrast, the soft chemical synthetic method involves the dissolution of Li and Mn compounds in aqueous solutions. Several soft chemical processes, including sol–gel, hydrothermal, and molten-salt techniques, have been applied to produce precursors with enhanced homogeneity, purity, and crystallinity. Solid-phase [37,38] and soft chemical methods employing sol–gel [39] and hydrothermal techniques [40,41] are common for precursor preparation in LTO-type LISs. Further details on precursor preparation for LISs can be found in Tables 1–3 and Figure 8 of reference [6]. Upon preparation of precursors, LISs are typically obtained through acid treatment to remove the Li ions (de-lithiation) while maintaining an imprinted three-dimensional structure. It is worth noting that the Li-ion intercalation process may be accompanied by the dissolution of Mn, resulting in deformation or possible collapse of the three-dimensional structure. Redox and ion exchange reactions, along with a composite of the two processes, have been reported to be the mechanisms for the intercalation/de-intercalation of Li ions in LISs. Detailed discussions on the acid treatment of precursors and their mechanisms are reported elsewhere [33,42,43,44,45,46]. Crosslinking of biopolymer-based granulated LISs to form well-defined composite materials is common [24,47,48,49,50,51,52,53] and can be achieved either before or after granulation, with variable outcomes. Crosslinking prior to granulation is anticipated to enhance the integrity of the LIS granules due to the mechanical stability of the crosslinked powdered sieve materials.

## 3. Granulation of LISs

In Section 2, the design of LISs from precursor preparation to intercalation of Li ions by ion exchange using an acid was introduced. This section will focus on the design processes to improve the structural integrity and performance of LISs. Direct Li extraction using nano-based LISs is considered a promising technology for Li recovery since LISs are generally considered non-toxic, cost-effective, and chemically stable [54,55]. Moreover, LISs have a high capability to selectively extract Li from a matrix that contains other ions like Na, Ca, and Mg. However, since LISs are usually available in powder form, higher pressure drops, and energy consumption impact their Li separation efficiency and limit their use in industrial large-scale applications. Thus, several methods like foaming, granulation, fiber formation, magnetization, and membrane formation have been developed to modify the structure and morphology of LISs to overcome this challenge [56]. Granulation is the generally accepted technology in nanoparticle modification through which LIS powder can form composite microspheres of various sizes and forms by bonding with the polymeric binding agents. The granulation of LISs affords the formation of modular inorganic–organic composite materials that comprise different metal-oxides embedded within a biopolymer scaffold. This process produces microspheres with good mechanical strength and easy adaptability to industrial column operation. While the current study focuses on biomaterial-based binders, some examples of literature are provided on the use of inorganic particles as binding agents for LISs.

### 3.1. Granulation of LISs Using Inorganic and Synthetic Organic Materials

**Figure 3:**
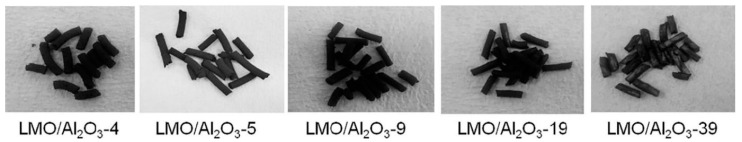
Pictures of LMO/Al_2_O_3_ composites (reproduced from [23] with permission).

Among the binders used for the granulation of metal based LISs, studies that report on the use of synthetic organic polymers, as well as inorganic materials, are prevalent in open literature and will be highlighted here. For example, Xiao et al. [57], Zhu et al. [58], and Yang et al. [59] evaluated the adsorption properties of LISs granulated with polyvinyl chloride (PVC) as the binder. Nisola et al. [60] fabricated and evaluated the Li uptake properties of macroporous foam composites made of polyvinyl alcohol (PVA). Similarly, Limjuco and coworkers [61] prepared H_2_TiO_3_ composite granules for efficient and continuous recovery of Li ions from liquid resources using PVA as the binder. Xiao et al. [62] prepared LMO-based granulated sieves with a polyacrylamide (PAM) binder via the inverse suspension polymerization method, resulting in composite microspheres with a diameter range between 0.3 and 0.7 mm. In another study, Jia and coworkers [63] prepared and evaluated the adsorption–desorption properties of LMO sieves granulated with polyacrylonitrile and polyvinylpyrrolidone binders. Epoxy resins have also been used as binders for granulating LISs and subsequent use for recovering Li ions from brine with good adsorption and recovery performance [57,58,64,65,66,67]. For instance, Liu et al. [66] and Zhang et al. [67] employed an amphiphilic epoxy-based binder (polyvinyl butyral) for granulation of LTO-sieves for the recovery of Li ions from brines of varying pH conditions. The use of mineral-based binders (e.g., alumina and silica) has also been reported in the literature. Hong et al. [23,68] employed alpha-alumina beads (AABs) and calcined mesoporous alumina (Al_2_O_3_) as immobilizers/binders to enhance the textural and surface properties of LTO-sieves for efficient recovery of Li ions from brine. The granulated LISs prepared by Hong and coworkers with AAB binder showed excellent Li ion recovery (8.87 mg g^−1^) from seawater with good cycling performance after up to 15 adsorption–desorption cycles. On the other hand, the LISs granulated from mesoporous alumina exhibited Li ion uptake capacity comparable to that of the corresponding powder material owing to the expanded surface area and porous structure imparted by the mesoporous γ-Al_2_O_3_ binder (cf. Figure 3). However, the use of an excessive amount of Al_2_O_3_ in the later negatively impacted the crystallinity of the LMO spinel structure and led to Mn dissolution during the regeneration process. The latter highlights the importance of maintaining an optimum ratio of LISs/binder in the composites for optimal performance of the granulated LIS adsorbents.

**Figure 4:**
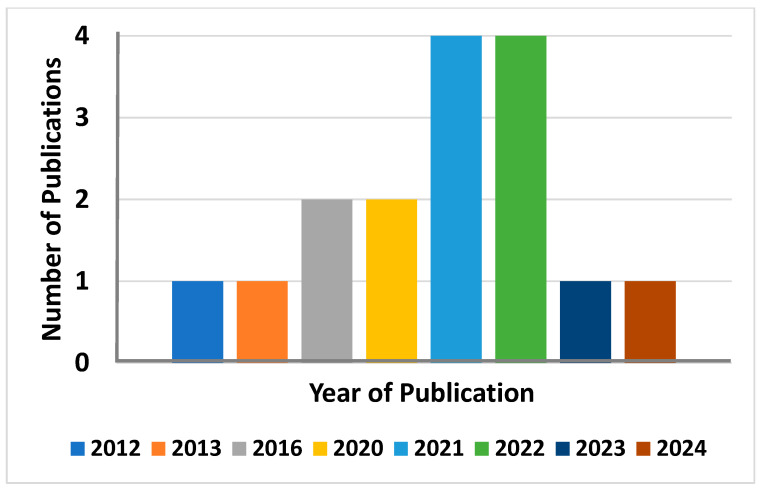
Bar chart showing the number of publications on the granulation of LIS using biopolymers in the last 10 years.

Contrary to several reports on the use of synthetic organic and inorganic materials as binders for the granulation of metal-based LISs, reports on the use of biopolymers as binders are limited in the open literature (cf. Figure 4) [24,47,48,49,50,51,52,69,70,71,72,73,74,75,76,77]. Additionally, there are currently no review articles that highlight the knowledge gaps on the use of biopolymers as binders for the granulation of LISs. Most recent studies have focused on the use of chitosan, alginate, agar, and cellulose as binders to prepare granulated LISs [24,47,48,49,50,51,52,69,70,71,72,73,74,75,76,77]. For instance, numerous studies [47,48,49,50,51,78] have reported on the use of chitosan in various forms (crosslinked and non-crosslinked) as a binder for the granulation of LISs. Similarly, other studies [58,59,60,61,62,63,64,65] have reported on the use of cellulose and its derivatives for the granulation of LISs. Li et al. [72], Koilraj et al. [73], Luo et al. [24], and Park et al. [74] reported on the use of crosslinked alginate for the granulation of Li-ion sieves, while Han et al. [76] and Chen et al. [77] reported on the use of agar. The non-availability of many research studies and review articles on the use of biopolymers as binders for the granulation of metal-based LISs highlights the need for a greater understanding of the knowledge gaps and limitations on the use of these abundant, nontoxic, environmentally friendly, and low-cost biomaterials in the Li extraction industry (cf. Figure 4). This review, therefore, intends to highlight the knowledge gaps on the use of biopolymers as binders for the granulation of metal-based LISs to catalyze further research into the use of these environmentally friendly materials in the Li industry. Section 3.2 discusses, in detail, the preparation of LIS granules using biomaterials as the binding agents.

### 3.2. Granulation of LISs Using Biopolymers

**Scheme 2:**
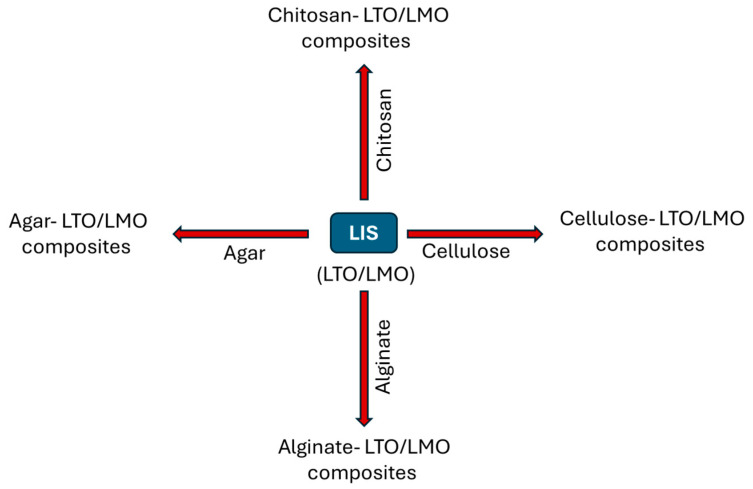
Schematic representation of the organic (biopolymer)–inorganic (LIS) composite materials captured in the review.

Biopolymers refer to renewable, biodegradable, and environmentally friendly polymers that are derived from animals, plants, and microorganisms [79,80]. They may be classified according to their origin (natural, synthetic, and microbial) and monomer units (e.g., polynucleotides, polypeptides, and polysaccharides) [80]. Biopolymers have gained wide interest in various research fields because of their structural diversity, imparted by the presence of diverse reactive functional groups. Interest in their use in the granulation of metal-based LISs relates to their abundance and other unique properties such as non-toxicity, low-cost, and biodegradability with reduced environmental footprint [81]. This review article is focused on the use of chitosan, cellulose, alginate, and agar as binders for the granulation of metal-based LISs to afford LMO-biopolymer and LTO-biopolymer composite materials (cf. Scheme 2). Further details on these selected biopolymers are highlighted below.

#### 3.2.1. Metal-Based LISs Granulated with Chitosan

**Figure 5:**
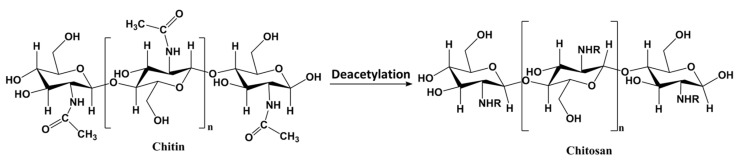
Structure of chitin and chitosan, where n represents the number of repeating units (R = H or acetyl groups according to the level of deacetylation).

Chitosan (CTS) is a natural biopolymer derived from chitin, which is found in the exoskeletons of crustaceans, fungi, insects, and some algae [82,83]. The structure of CTS is shown in Figure 5 and comprises repeating units of glucopyranose rings with amine (–NH_2_ or –NHCOCH_3_) groups at the C-2 unit. The interest in the use of CTS as a binder for the granulation of LISs relates to its good solubility at mildly acidic conditions, low cost, biodegradability, non-toxicity, and hydrophilic nature.

**Scheme 3:**
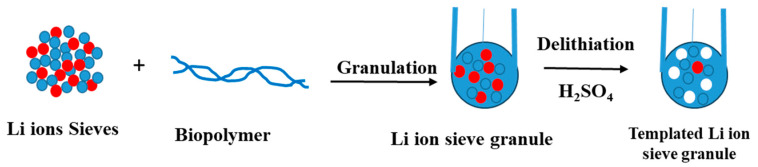
Granulation of LISs using a Biopolymer Binder to Composite Adsorbent Material.

Several research groups have reported on the preparation and utility of granulated metal-based LISs using CTS as the binding agent. Hong et al. [47] prepared mesoporous CTS-granulated LISs using Li_1.33_Mn_1.67_O_4_ precursor, where the precursor was prepared through a solid-state synthetic method. The solvation of CTS was achieved by dissolving 6 g of the CTS binder in 4% lactic acid at 80 °C. The LMO-based ion sieves (4 g) were added to the CTS solution (100 mL) at the 4% (*w*/*v*) ratio to form a paste, followed by granulation into cylindrical-shaped composite microcapsules using an extruder, as depicted in Scheme 3. De-lithiation of the prepared granules with 0.2 M sulfuric acid revealed a topotactical Li ion extraction at 93% efficiency. Characterization of the prepared granules via FTIR spectroscopy supported the crosslinking of CTS with the sulfate anions (SO_4_^2−^) in sulfuric acid following de-lithiation. De-lithiation of the granules revealed changes in the morphology of the granulated LISs due to changes in the textural properties of the binder and/or the granules. However, the adsorption capacity of the LIS was retained at ~10 mg/g with increased mechanical stability following the granulation process.

In another report, Ryu et al. [48] studied the recovery of Li ions from seawater using a continuous flow adsorption column packed with CTS-granulated LMO-based microcapsules. The synthetic strategy for the granulated LISs was similar to that reported by Hong and coworkers [47] (cf. Scheme 3). The CTS paste was extruded into 0.7 mm cylinder-shaped granules, whereas the materials were characterized by FTIR spectroscopy in agreement with the results of Hong et al. [47]. Ryu and coworkers evaluated the effects of Li-ion concentration, contact time, and recyclability of the prepared CTS–LMO granules. A good Li ion removal efficiency and selectivity were reported, where the adsorption capacity of the granules was recorded at ~54.65 mg/g. The regeneration performance was evaluated using H_2_SO_4_ (SO_4_^2−^) and revealed a slight decrease in the adsorption capacity of the composite granules for Li ions after three adsorption–desorption cycles. Similarly, the Li-ion recovery capacity decreased slightly from 88 to 85% during the third adsorption–desorption cycle. The trends in the adsorption and recovery capacities were attributed to Mn ion loss (7–9%) and the subsequent collapse of the CTS–LMO granule three-dimensional structure.

**Figure 6:**
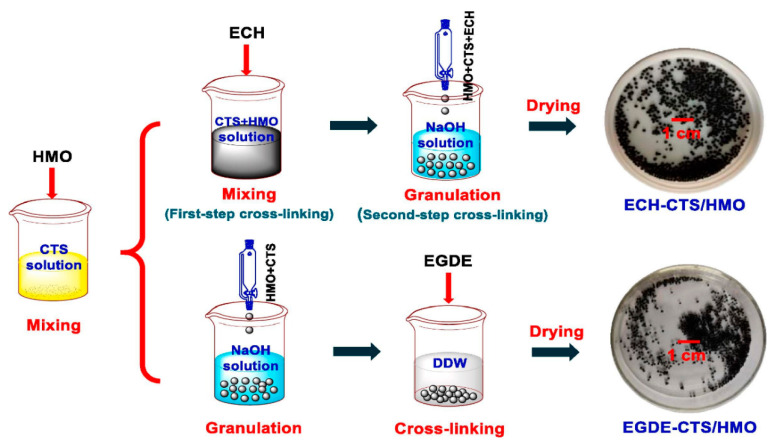
Fabrication of ECH–CTS/HMO and its difference from the preparation of EGDE–CTS/HMO (reproduced from [49] with permission). the black arrows depict the reaction process, while the red arrows depict reagent addition.

In another study, Ding et al. [49] prepared granulated LMO-based LISs, with Li_4_Mn_5_O_12_ as the precursor and CTS as the binder, using a novel crosslinking strategy. The Li_4_Mn_5_O_12_ precursor was de-lithiated with HCl to obtain H_4_Mn_5_O_12_ (HMO) for subsequent granulation and crosslinking processes. The crosslinking strategy employed by Ding et al. [49] to prepare the HMO/CTS is different from the study reported by Ryu et al. [48] and Hong et al. [47]. In the latter, crosslinking of the LMO/CTS composite materials was achieved using polyatomic (sulfate) anions upon granulation and de-lithiation of the LISs. In the present study, the use of epichlorohydrin (ECH) and ethylene glycol diglycidyl ether (EGDE) as crosslinker molecules was aimed at improving the three-dimensional structure and introducing better mechanical strength of the LIS granules. The fabrication of the crosslinked HMO LISs is schematically presented in Figure 6. Crosslinking of the HMO sieves using ECH as a crosslinker was performed in two steps, both prior to- and post-granulation. In the first step, 3.0 g of the LISs were added to a 3.0 wt% CTS solution in a glacial acetic acid solution and mixed thoroughly. The ECH crosslinker molecule was added in situ at equimolar concentration with respect to CTS, and the mixture was allowed to react for 2 h at 323 K. This was followed by slowly dropping the dark brown suspension into a 1 M NaOH solution to form spherical granules, and the reaction was allowed to continue at 323 K for another 5 h for a second-step crosslinking. The formed spherical adsorbents were washed with ethanol and deionized water in sequence before use. For the EDGE crosslinked granules, the crosslinking was achieved upon granulation of the HMO LISs, where 3.00 g of LISs were dispersed in 100 mL of 3.0% CTS. The mixture was then dripped into 1 mol/L NaOH to form spherical CTS–LIS granules. After washing to neutral pH, these materials were placed in 100 mL of deionized water and crosslinked with EDGE at a mass ratio of EDGE/CTS = 2 at 318 K for 9 h.

**Figure 7:**
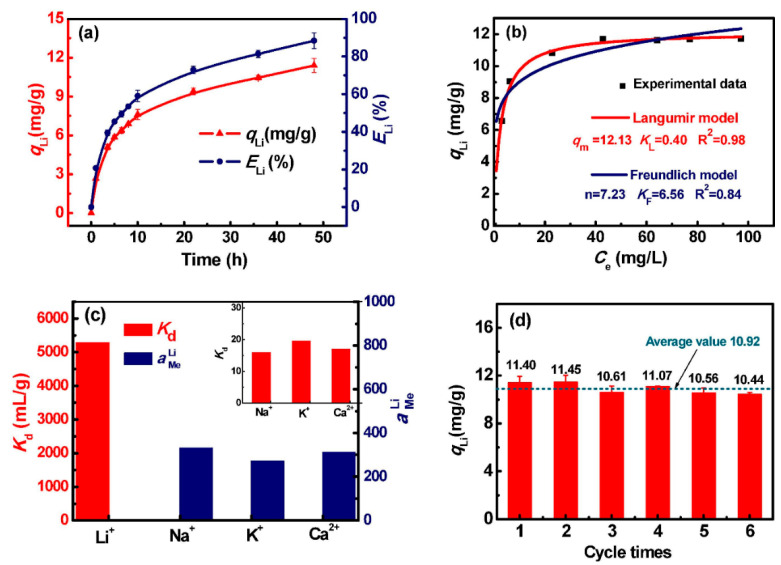
Recovery of Li-ion from geothermal water by ECH–CTS/HMO: (**a**) Variations of adsorption capacity and recovery rate with time (S/L = 1 g/250 mL, pH = 12, T = 333 K); (**b**) adsorption isotherms and fitted curves with different isotherm models; (**c**) adsorption selectivity; (**d**) cyclic stability (reproduced from [49] with permission).

Ding and coworkers’ strategy to de-lithiate LMO sieves prior to granulation and crosslinking was meant to prevent agglomeration of the non-de-lithiated LMO particles, which may, in turn, lead to the formation of nonhomogenous granules affirmed by SEM and particle size analysis results (cf. Figures S1b and 4a in reference [49]). The HMO–CTS materials prepared by Ding et al. [49] are expected to exhibit superior physicochemical properties compared to the LMO–CTS composites reported by Hong et al. [47] and Ryu et al. [48] This is because crosslinking of HMO and LMO LISs is anticipated to produce composites with variable physicochemical properties due to variable reaction mechanisms of CTS with the H- and Li-type sieves. Ding et al. [49] reported that mass loss due to Mn dissolution, as well as the adsorption capacity of the granules, increased with increasing LIS content, where the ECH crosslinked granule displayed higher Mn dissolution, relative to the EGDE crosslinked granule. TG curves of the ECH and EGDE crosslinked granules revealed that both materials had high chemical stability. The later supports the successful crosslinking of the LIS granules with a concomitant decrease of hydrophilicity relative to pristine CTS, in agreement with contact angle results. Adsorption studies with the granulated composite LISs revealed that the two-step ECH crosslinked granules display superior adsorption/desorption characteristics relative to the EGDE crosslinked granules (crosslinked after granulation). Due to this reason, the ECH crosslinked material was used for further studies. Adsorption studies with the ECH crosslinked granule reveal that the adsorption capacity increased sharply initially and was 11.40 mg/g after 48 h, where the maximum adsorption capacity according to the Langmuir adsorption model was 12.13 mg/g, as shown in Figure 7. The Li-ion recovery rate was approximated to be 88% based on the use of 1 g of LISs per 250 mL of the adsorbate. When the granulated material was further used for Li-ion recovery from geothermal water at a solid-to-liquid ratio of 4 g/L, the adsorption capacity and recovery rate were 11.4 mg/g and 88.42%, respectively. The authors also reported that the ECH crosslinked granules revealed a very minimal adsorption capacity loss after six adsorption–desorption cycles (Figure 7d), as well as high selectivity for Li-ions in the presence of other contaminants (Figure 7c), where the separation factor between Li and all other ions was greater than 270 (cf. Figure 7). This study demonstrates that crosslinking of the biopolymer before granulation leads to greater mechanical, chemical, and thermal stability.

**Figure 8:**
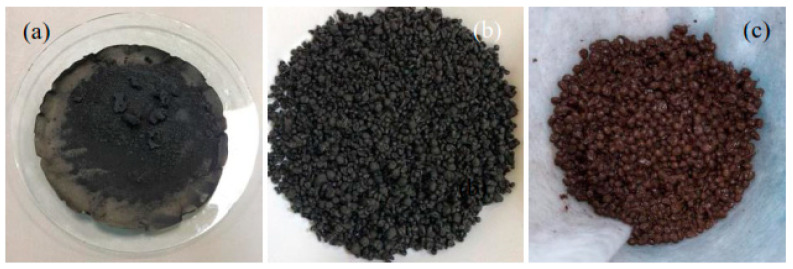
Three forms of adsorbents: (**a**) LMO before granulation with chitosan, (**b**) granulated LMO, and (**c**) de-lithiated LMO (reproduced from [50] with permission).

Yüksel Özşen and Kahvecioğlu [50] studied the recovery of Li ions from water resources using LMO-based LISs granulated with CTS, followed by crosslinking with ECH to enhance their adsorption yield, stability, and reusability. The granulated LISs were prepared using a 3% CTS solution in 2% acetic acid. 2 g of the LISs were added to the CTS solution with mixing until a homogenous mixture was obtained, where the CTS–LISs mixture was extruded into a 1 M NaOH solution to obtain the CTS–LIS granules (cf. Figure 8). Contrary to the strategy employed by Ding et al. [49], crosslinking of the LISs prepared by Yüksel Özşen and Kahvecioğlu [50] was achieved in the presence of NaCl and KOH. It is believed that the role of the salts is to maintain the structural integrity of the beads by modulating their interaction with water through the “salting out” effect in agreement with the study of Qilei Zhang et al. [84]. Five grams of the granules and 0.075 g of NaCl were added to 75 mL of water containing 0.35 mL of ECH with continuous mixing. This was followed by the slow addition of 2 mL pure water containing 0.3 g of KOH, and the mixture was stirred for 16 h at 25 °C. The resulting granules were dried at 70 °C overnight, followed by characterization via SEM, XRD, and BET studies. The SEM results show that the granulated adsorbent was mesoporous, with the LISs uniformly distributed in the binder matrix. Adsorption studies revealed that the optimum conditions for Li-ion uptake by the granulated LISs were pH 12, an adsorbent dosage of 4 g/L, and an equilibrium time of 10 h. The Li removal efficiency during the first adsorption cycle was 90%, while recovery was 95%. However, during the second cycle, the Li removal efficiency and recovery decreased by 80% and 50%, respectively. The authors attributed the efficiency drop to structural deformity of the granulated materials upon treatment with HCl during the de-lithiation process, as described above. The later was affirmed by a decrease in the size of the granulated LISs. The granules prepared by Yüksel Özşen and Kahvecioğlu [50] were less stable compared to similar materials prepared by Ding et al. [49]. This difference in stability is due to differences in the crosslinking strategies. Crosslinking prior to granulation is anticipated to produce granules with improved mechanical stability in accordance with the results of Ding et al. [49].

**Figure 9:**
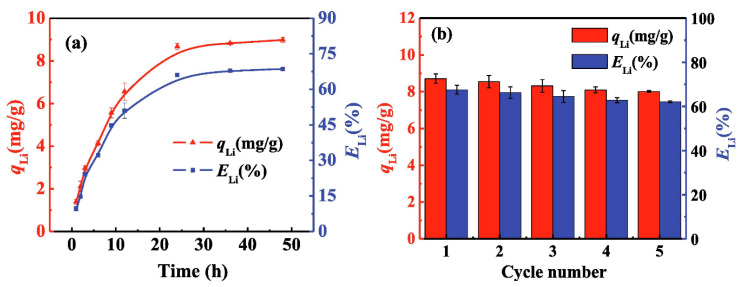
Adsorption of Li-ions from geothermal water by crosslinked CTS/HMO at a solid-to-liquid ratio of 1:200 and pH 12. (**a**) Variations of adsorption capacity and efficiency with time; (**b**) cycle stability (reproduced from [51] with permission).

Wang et al. [51] granulated different types of LMO sieves, such as LiMn_2_O_4_, Li_1.66_Mn_1.66_O_4_, and Li_4_Mn_5_O_12_, using CTS as the binder at a mass ratio of CTS/LMO = 3:2 for the selective recovery of Li ions from geothermal water. The granules were prepared using a 3% CTS solution in 2% acetic acid. 2 g of the LMO were added to the CTS solution while stirring until a homogenous mixture was obtained. The homogenous mixture was extruded into a 1 M NaOH solution to form the LIS granules. The authors noted that the hydrophilicity of CTS enhanced its hydration in acidic media, thus resulting in the degradation of the CTS domain of the granules through dissolution. Therefore, crosslinking was carried out post-granulation using various crosslinkers, where the crosslinker/CTS mass ratios were as follows: EGDE/CTS = 2, glutaraldehyde (GLY)/CTS = 2, and ECH/CTS = 1, and it successfully resolved the degradation of the CTS domain of the granules. Crosslinking has been reported to increase the thermal and chemical stability of biopolymers [79,85,86]. Crosslinking of the granulated LISs with EGDE resulted in reduced CTS mass loss due to dissolution from 27.65% to 0.6%. The authors compared the effects of crosslinking with other crosslinkers, such as ECH and GLY, and reported that crosslinking with EGDE improved the mechanical and chemical stability of the granules, as shown by greater resistance to mass loss due to CTS dissolution. Studies on the optimization of adsorption and desorption conditions using the granulated LISs reveal that though an increase in hydrogen ion concentration and temperature increased the desorption efficiency of the granulated LISs, it negatively impacted chemical and mechanical stability. Therefore, the optimum conditions for the adsorption process were pH = 12 and room temperature, while 0.25 M HCl was most suitable for the desorption process. The authors applied the obtained optimum conditions for the adsorption of Li ions from geothermal water and reported that adsorption equilibrium was attained within 24 h, where the equilibrium adsorption capacity was 8.98 mg/g, as shown in Figure 9. The authors also reported a 1.1% loss in adsorption capacity after five adsorption–desorption cycles, where the selectivity coefficient (K_d_) for Li ions was 1113.27 mL/g, suggesting high selectivity for Li ions by the granulated LISs materials (cf. Figure 9). Contrary to the results of Wang et al. [51], the greater stability of the ECH-crosslinked granules reported by Ding et al. [49] is accounted for by the use of a two-step crosslinking strategy, where crosslinking was performed before and after the granulation process. Despite the use of NaCl and KOH by Yüksel Özşen and Kahvecioğlu [50], the produced LIS granules were not more stable relative to materials produced by Ding et al. [49]. The latter highlights the importance and effectiveness of introducing crosslinking prior to the granulation process in supporting the chemical and mechanical stability of granulated LISs.

Recepoğlu et al. [78] reported on the granulation of hydrometallurgically synthesized LISs using crosslinked CTS as the binder. Granulation was performed using a CTS/LMO ratio of 3:2, where 3 g of CTS was dissolved in 97 mL of 2% glacial acetic acid at room temperature. This was followed by the addition of 2 g LMO to the 3% CTS solution with blending until a homogeneous mixture was obtained. A needle-tipped syringe was used to drop the CTS/LMO mixture into a 1 M NaOH solution to obtain the spherical CTS/LMO granules. Excess NaOH was removed by washing the granules with deionized water till the pH of the washing water was 7. The LIS granules were dried at 60 °C for 12 h before further use. Crosslinking of the LIS granules via epichlorohydrin as the crosslinker was achieved in the presence of NaCl and KOH at 25 °C for 16 h, followed by washing with deionized water to remove unreacted reagents. De-lithiation of the granulated LISs was performed using 0.25 M HCl to obtain the CTS/HMO granules. SEM, XRD, FTIR, and BET techniques were used to evaluate the physicochemical properties of the granulated CTS/LISs biocomposites. The adsorption performance of the granulated LIS materials was elucidated at pH 2–12, 25 °C using various concentrations (10, 25, 50, 75, and 100 mg/L) of LiCl as adsorbate, various time intervals (0, 5, 10, 15, 20, 30, 45, 60, 90, 120, 240, 360, 480, 1440 min) and adsorbent dosages of 1.0, 2.0 and 4.0 g/L. The stability of the granulated LISs biocomposites was also evaluated through a two adsorption–desorption cycle experiment using 0.25 M, 0.5 M, and 1.0 M concentrations of HCl solutions and 4 g/L adsorbent dosage. Results obtained from the study reveal that maximum adsorption performance was achieved with a 4 g/L adsorbent dosage and pH 12 at 25 °C. The authors reported that the adsorption of Li ions by the granulated LISs biocomposites was driven by physical forces such as weak electrostatic and van der Waal’s interactions. Furthermore, results from stability studies reveal that 0.25 M HCl is best suitable for the desorption of Li ions from the granulated LISs biocomposites, where there was a significant drop in the adsorption and desorption performance of the biocomposite granules due to structural deformation. The synthetic strategy employed in this study is very similar to the one reported by Wang et al. [51] and Yüksel Özşen and Kahvecioğlu [50], where both authors reported poor cycling performance of the granulated LIS biocomposites in agreement with the report of this study by Recepoğlu et al. [78] Contrary to the reports of Recepoğlu et al. [78], Wang et al. [51] and Yüksel Özşen and Kahvecioğlu [50], Ding et al. [49] reported better cycling performance and greater stability of the ECH-crosslinked granules which may be attributed to the use of two-step crosslinking strategy, where crosslinking was performed before and after the granulation process.

#### 3.2.2. Metal-Based LISs Granulated with Cellulose

**Figure 10:**
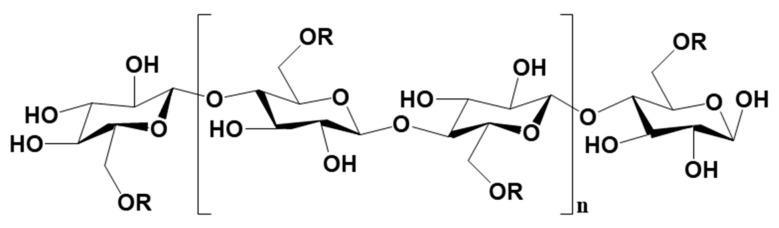
Molecular structure of cellulose, carboxymethyl cellulose, and hydroxyethyl cellulose (where n represents the number of repeating units, R = H for cellulose, –CH_2_COONa for carboxymethyl cellulose and CH_2_CH_2_OH for hydroxyethyl cellulose).

Cellulose (CLS) (cf. Figure 10) is an abundant, environmentally friendly, and renewable biopolymer [79,87,88]. It may be derived from both plants and animals and, just like CTS, is composed of D-anhydro glucose units linked by β-l, 4-glucosidic bonds that exist between the carbon atoms C1 and C4 of adjacent glucose units. The only difference between CTS and CLS is the functionality at C-2 of the anhydro glucose unit. The inherent intra/intermolecular hydrogen bond networks of CLS biopolymer afford the formation of partially crystalline and amorphous regions, which limits its solubility [89]. However, studies [69,86,90] have shown that the use of mixed solvent systems ranging from alkali/urea solutions [91,92,93] to ionic liquids (ILs) [94,95,96] affords the preparation of CLS hydrogels for various industrial applications. Similarly, the synthesis of various derivatives of CLS, such as carboxymethyl CLS and hydroxyethyl CLS, improves its hydration properties, thus enhancing its solubility in conventional solvents. The interest in the use of CLS as green matrices in various applications is related to its hydrophilicity, biodegradability, and amenability [97]. This section of the review highlights progress in the application of CLS and its derivatives as binders for the granulation of LISs.

**Figure 11:**
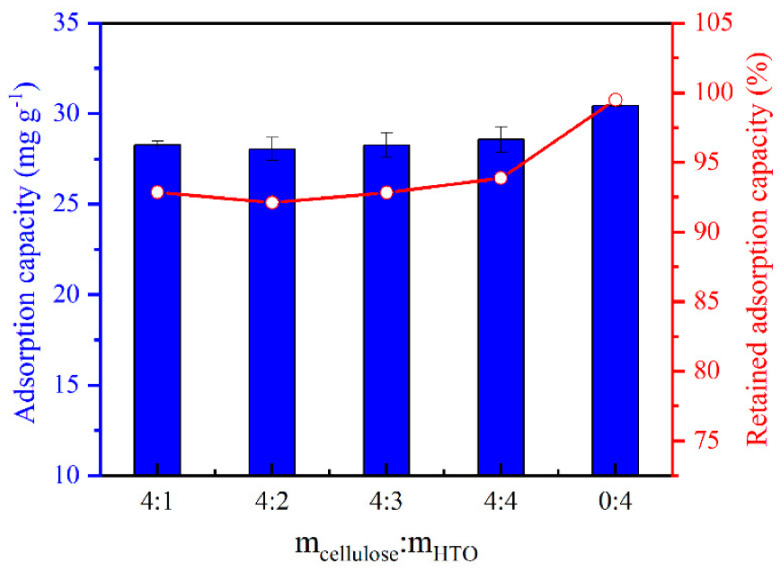
Li-ion adsorption capacity of HTO/CA with different HTO loadings and respective % qe retained by HTO in CA for Li^+^ (pH = 10.25; V = 160 mL; m ≅ 100 mg; C0 = [Li^+^] ≅ 50 mg L^−1^) (reproduced from [69] with permission).

Qian et al. [69] developed a series of H_2_TiO_3_/CLS aerogels (HTO/CA) with a porous network for the selective recovery of Li ions from seawater. The granules were prepared by dissolving CLS in an ionic liquid [Bmim]Cl at 90 °C for 1 h. Subsequently, the LISs (HTO)–CLS mixture with variable CLS to HTO ratio (4:0, 4:1, 4:2, and 4:3) was prepared and thoroughly mixed to afford homogeneity. The resulting mixture was poured into a mold, and air bubbles were removed with the aid of a vacuum-drying oven, followed by coagulation in an ethanol bath for 24 h. The obtained hydrogel was washed with deionized water, pre-frozen at −12 °C, and finally freeze-dried under high vacuum conditions for 48 h. The HTO/CA sorbents were characterized using FTIR, XRD, SEM–EDS, contact angle, and nitrogen adsorption studies. Nitrogen adsorption results revealed a higher surface area for the HTO/CA (60.38 ± 2.65 m^2^/g) relative to HTO powder (22.77 m^2^/g), while XRD results noted similar diffraction patterns for HTO powder and HTO/CA [38,39,61] but decreased peak intensities for HTO/CA. The adsorption capacities of the aerogels with different HTO/CA mass ratios were tested, and it was observed that adsorption capacity increased slowly with an increase in the loading of HTO powder, as shown in Figure 11. The LIS aerogel with the highest HTO loading level (4:4) exhibited a slightly higher adsorption capacity of 28.58 ± 0.71 mg/g relative to the aerogels with other loading levels. The results show that following granulation, this HTO/CA material retained >90% of the adsorption capacity of HTO powder (cf. Figure 11). Further adsorption studies revealed that HTO/CA exhibited a 69.93 ± 0.04% Li removal from seawater with a high affinity for Li ions relative to other ions present in the matrix. Regeneration studies using LiCl solution showed that the Li adsorption capacity of HTO/CA changed slightly after the first five adsorption–desorption cycles, with the final adsorption capacity being 22.95 mg/g.

**Figure 12:**
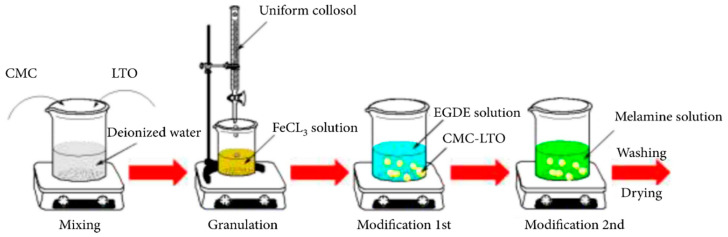
Schematic presentation of CMC–LTO–EGDE–ME microsphere preparation procedure (reproduced from [52] with permission).

In another study, Xie et al. [52] synthesized spherical composite LISs made of CMC-CLS, EGDE, melamine (ME), and LTO-based sieves with Li_2_TiO_3_ as the precursor. The multi-step crosslinked granules were made as follows: 1.5 g of Li_2_TiO_3_, 1.5 g CMC, and 100 mL of double deionized water (DDW) were added to a beaker and stirred at ambient temperature for about 6 h with sonication for several minutes to obtain a homogeneous mixture. The mixture was added dropwise into 100 mL of 2 wt% FeCl_3_ solution with stirring to obtain the CMC–LTO granules which were washed severally with DDW to remove unreacted chemicals. Crosslinking was achieved by adding the granules to 2 wt% EGDE solution and allowed to react for 6 h at 318.15 K, followed by washing with DDW. The obtained CMC-LTO-EGDE LIS composite granules were further crosslinked with ME by placing them in 2 wt% ME solution and mechanically stirred at 318.15 K for 6 h to afford a complete crosslinking reaction (cf. Figure 12). The composite granules were filtered, washed with DDW, and dried under vacuum at 318.15 K for 24 h. The synthesized composite granules were characterized by FTIR and SEM methods. Adsorption studies with the LIS granules showed that optimum conditions for the uptake of Li ions by the composite granules were pH 12 and 24 h equilibration time, where the uptake capacity was 12.02 mg/g. Desorption studies at variable concentrations of HCl as the eluent revealed that titanium loss increased with increasing acid concentration. Further studies on the reusability of the LIS granules reveal that there was a marginal decrease in the uptake capacity of LIS granules after each adsorption–desorption cycle. The materials prepared by Xie et al. [52] and Qian et al. [69] offer different strategies for the design of LISs with different adsorption–desorption performances. Xie and coworkers employed a porous aerogel structure to improve the adsorption performance of the LISs. By contrast, Qian et al. [69] applied a multi-step crosslinking strategy to afford multiple adsorption binding sites, as well as to enhance the mechanical stability of the produced granules.

**Figure 13:**
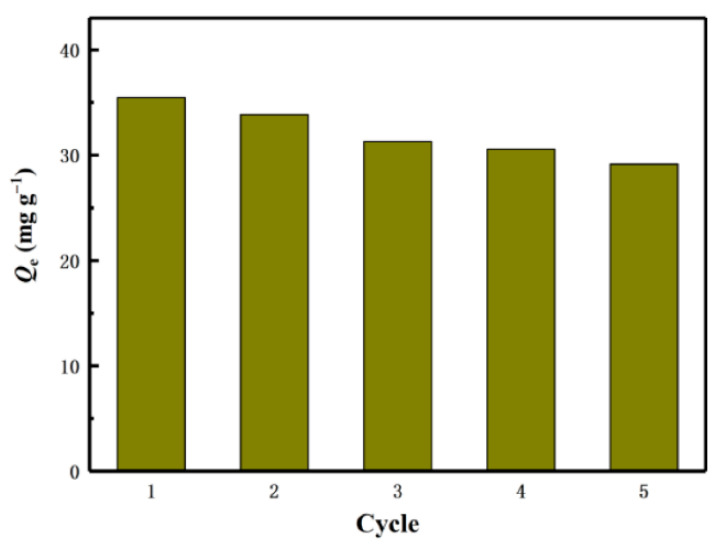
Regeneration of H_2_TiO_3_ five cycles (reproduced from [70] with permission).

Zhang et al. [70] reported the preparation of high-specific surface LISs templated by bacterial CLS for the selective adsorption of Li ions. The templated LISs were synthesized as follows: Bacterial CLS (B-CLS) hydrogel was added to deionized water for 15 min to enhance swelling, followed by freezing with liquid nitrogen and freeze-drying to obtain a B-CLS aerogel network structure. The B-CLS aerogel network was soaked in titanium ethoxide solution for 2 h, rinsed with ethanol and ultrapure water about 4 to 5 times, followed by stirring in ultrapure water for 2 h, and washed again with deionized water. Finally, the obtained TiO_2_/B-CLS material was dried in the oven. The prepared templated LIS was characterized through FTIR, SEM, nitrogen adsorption, and XRD studies. Adsorption studies with the B-CLS templated LISs revealed that uptake was favored at alkaline pH conditions (pH 11), where increasing time favored the adsorption process till about 200 min. A maximum adsorption capacity of 35.45 mg/g was achieved at an equilibrium time of 6 h, which represented 80% of adsorption efficiency. The authors also reported that the B-CLS templated LISs display good selectivity for Li ions as well as strong regeneration ability, where the adsorption capacity remained above 82% of the initial value after five adsorption–desorption cycles, as shown in Figure 13. The synthetic strategy employed by Zhang et al. [70] is similar to the study of Qian et al. [69], where the notable difference between the two synthetic strategies relates to the use of an ionic liquid ([Bmim]Cl) for the dissolution of CLS by Qian et al. [69].

**Figure 14:**
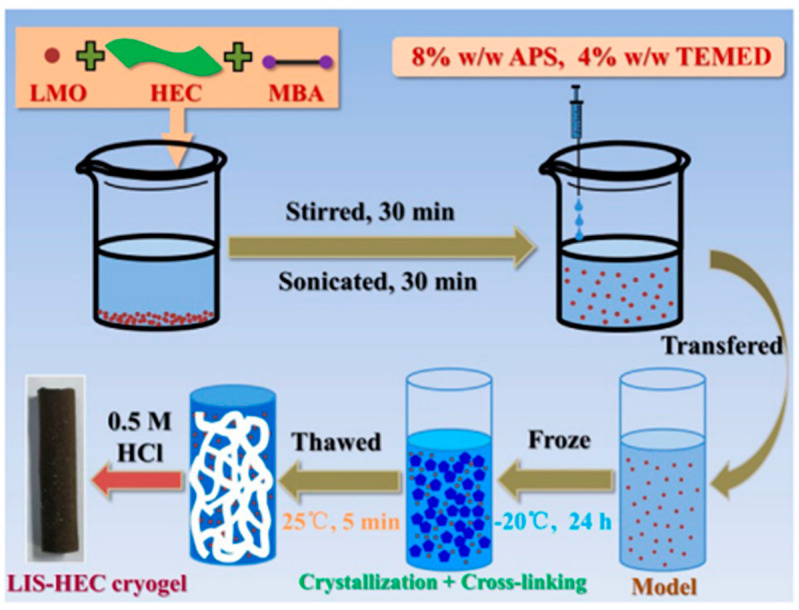
Schematic illustrating of the preparation process for LIS–HEC cryogels (reproduced from [71] with permission).

In a separate study, Liu et al. [71] reported on the preparation and characterization of LISs embedded in a hydroxyethyl CLS cryogel (LIS–HEC) for the continuous recovery of Li from brine. The LIS–HEC cryogel was prepared as follows: 1.75 g (3.5% *w*/*w*) of HEC and 0.75 g (1.5% *w*/*w*) of N,N’-methylenebis (acrylamide) (MBA) were dissolved in deionized water with mixing and sonication to enhance bubbles elimination. Various amounts (0.5, 1.0, 1.5, 2.0, and 2.5 g) of the LISs (LMO) were added to the viscous HEC solution at 0 °C, and the resulting mixture stirred for 10 min, followed by sonication for 10 min, respectively. The stirring and sonication sequence was repeated thrice, followed by the dropwise addition of ammonium persulphate (APS) and 4 wt% N,N,N’,N’-Tetramethylethylenediamine, (TEMED) solutions to the mixture. The resulting mixture was injected into a cylindrical mold with a 10 mm inner diameter, cooled to −20 °C, and held for 24 h. The mixture was thawed at room temperature and rinsed with deionized water to obtain an LMO–HEC cryogel. The LIS–HECs were obtained via treatment of the LMO–HEC with 0.5 M HCl and referred to as LIS–HEC–N, where N in the LIS–HEC–N represents the LIS loading (wt%), as schematically represented in Figure 14.

**Figure 15:**
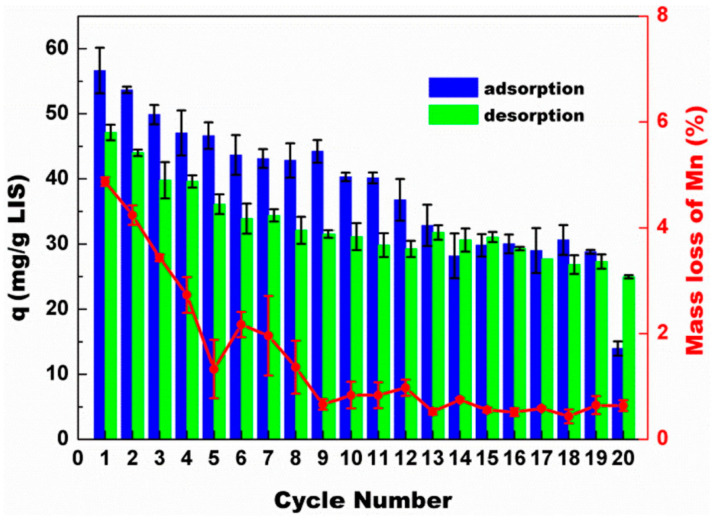
Adsorption–desorption cycling performance of the LIS–HEC-40 cryogel (C0 = 50 mg/L, pH = 9.51) (reproduced from [71] with permission).

The physicochemical, morphological, thermal, and textural properties of the synthesized composite cryogels were characterized using XRD, SEM, TGA, and BET analysis. Results obtained from batch adsorption studies with LiCl show that alkaline pH conditions (pH 9.51) favored the adsorption of Li ions by the composite materials, where the adsorption capacity of LIS powder was 30.5 mg/g. For the LIS–HEC, the results reveal that adsorption capacity increased monotonically with increasing LIS loading up to 80% loading, before it dropped slightly at 100 wt% loading. The authors attributed the drop in adsorption capacity of LIS–HEC-100 to the agglomeration of LIS particles on the surface of the composite cryogel, suggesting a collapse and/or inaccessibility of the templated active binding sites. The adsorption results also revealed that the uptake capacity of LIS–HEC cryogel did not change with increasing loading of LISs but was at an average of 47.8 mg/g. Similarly, results from isotherm studies at pH 9.51 indicate that the adsorption capacity of LIS powder was 44.78 mg/g, while LIS–HEC had a higher uptake capacity of 54.23 mg/g. These results show that the cryogel skeleton did not negatively impact the adsorption properties of LISs particles in the granulated adsorbents. Furthermore, adsorption studies with an artificial brine and seawater show that the adsorption capacity was 10.64 mg/g for brine and 2.46 mg/g for seawater, where the composite cryogel demonstrated good selectivity for Li ions in both adsorbate systems. Dynamic adsorption studies with the LIS–HEC cryogels revealed that a higher flow rate decreased the time required for attaining adsorption equilibrium, with the consequence of decreased adsorption capacity. The adsorption capacities per unit time of the adsorbents were 22.36, 39.45, 41.67, and 60.39 mg/g/h for flow rates of 1.0, 2.0, 3.0, and 4.0 mL/min, respectively. Reusability studies via multiple adsorption–desorption cycles indicate that the adsorption capacity of the granulated cryogel decreased by 49.11% after 19 cycles, where the desorption capacity followed the same trend. The decline in adsorption capacity was gradual during the first 10 cycles, where the tenth adsorption capacity was 71.25% of the initial value. From the 12th cycle, the adsorption capacity was stable till a sharp decrease was observed during the 20th cycle. Similarly, Mn loss showed the same trend, which indicates that the main reason for the attenuated adsorption capacity was the loss of Mn ions. Reusability results for the LIS–HEC cryogels are presented in Figure 15, where the adsorption–desorption trends show great promise for the HEC cryogel-immobilized LISs. The synthetic strategy employed by Liu et al. [71] and Xie et al. [52] are similar. Both authors crosslinked the prepared granules, though the crosslinking strategies were different. For example, Liu et al. [71] crosslinked CLS before granulation, whereas Xie et al. [52] crosslinked CLS during and after the granulation process. The results obtained by Xie et al. [52] further affirm the importance of crosslinking before granulation, in agreement with the study by Ding et al. [49].

#### 3.2.3. Metal-Based LISs Granulated with Alginate

**Figure 16:**
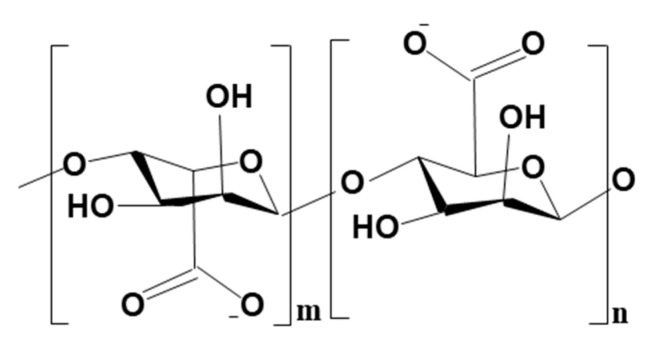
Molecular structure of alginate showing the M and G-blocks.

Alginates (ALGs) are soluble polyanion biopolymers that are biodegradable, non-toxic, and non-irritant in nature. They may be extracted from brown marine algae and bacteria, where α-l-guluronic (G) and β-d-mannuronic (M) are the acid residues (cf. Figure 16). ALGs exist commercially, mainly in calcium and sodium salt forms. The species of algae and the G/M ratio, along with their distribution along the biopolymer chain, influence the properties of the ALG that is extracted. Their monosaccharide sequencing and enzymatic controlled reactions make ALGs an important biopolymer group for diverse biological and biomedical applications [98,99,100]. This section of the review highlights the application of ALG as binders for the granulation of LISs.

**Figure 17:**
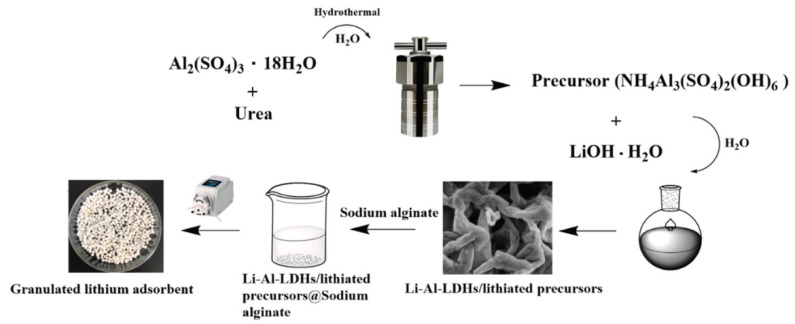
The synthetic route of granulated lithium adsorbents (reproduced from [24] with permission).

Luo et al. [24] reported the extraction of Li ions from Salt Lake brines using porous composite LIS mixtures of Li–Al–LDHs (layered double hydroxide) and NH_4_Al_3_(SO_4_)_2_(OH)_6_ crosslinked with sodium ALG as the crosslinker and binding agent. The reaction for the granulation of the composite LISs proceeded as schematically shown in Figure 17. The lithiated LIS precursors were added to a solution of ALG prepared by dissolving 2.0 g of ALG in 100 mL of deionized water. The resulting mixture was dripped slowly into a 4 wt% CaCl_2_ solution and held for 24 h for complete crosslinking [101]. The granulated LISs were washed with deionized water until the unreacted chemicals were completely removed.

**Figure 18:**
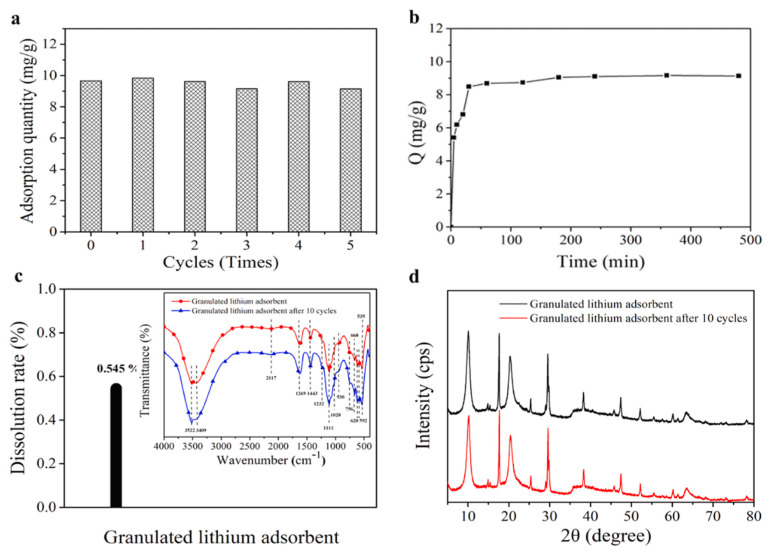
Lithium extraction from East Taigener Salt Lake brine. (**a**) Adsorption quantity change of granulated adsorbents after five adsorption–desorption cycles; (**b**) adsorption kinetic curve; (**c**,**d**) dissolution rate of granulated lithium adsorbents, structural change characterized by IR spectroscopy and XRD before and after 10 adsorption–desorption cycles (reproduced from [24] with permission).

The precursors and the granulated materials were characterized by FTIR spectroscopy, XRD, XPS spectroscopy, SEM, TEM, thermal analyses, particle size analysis, and BET surface area. Some of the results are presented in Figure 18. The adsorption properties of the granulated LISs were evaluated under the following conditions: adsorbate—LiCl, adsorbate concentration: 500 mg/L, oscillation speed: 200 rpm, temperature: 25 °C, time: 5–780 min, and pH: 4–10. According to Figure 18a,b, Li uptake by the granulated adsorbents increased initially to a maximum at pH 6 (9.66 mg/g) before it started decreasing. Furthermore, the results show that the concentration and temperature of Li solution had effects on the adsorption capacity of the granulated LISs, whereas the presence of competing ions like NaCl, MgCl_2_, Na_2_SO_4_, and KCl in the brines had minor effects on the selectivity of the LISs for Li ions. The adsorption properties of the granulated LISs were further tested with East Taigener Salt Lake brine. Results show that adsorption equilibrium was reached within 180 min, where there was no decline in adsorption capacity (9.16 mg/g) after five adsorption–desorption cycles. Mass loss due to dissolution of the granulated LISs was 0.54% after 10 adsorption–desorption cycles, suggesting that the structural and chemical integrity of the granulated LISs was not compromised after 10 cycles. The structural and chemical resilience was confirmed by FT-IR spectroscopy and XRD analysis, as depicted in Figure 18, where the signature peaks of granulated LISs before and after 10 adsorption–desorption cycles are shown.

**Figure 19:**
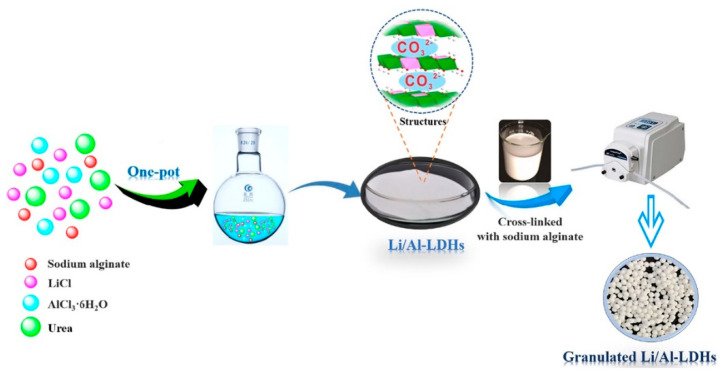
Synthetic route of granulated Li/Al–LDHs composites(reproduced from [72] with permission).

A similar study by Li et al. [72] reported the synthesis of granulated Li/Al–LDH adsorbents for the recovery of Li ions from simulated and real Salt Lake brines. The adsorbent was prepared by a one-pot dissolution method where LiCl (21.20 g), AlCl_3_·6H_2_O (40.20 g), and urea (100.01 g) were dissolved in 50 mL of deionized water at a 3:1:10 molar ratio and stirred vigorously. Subsequently, 2.12 g of Sodium ALG was dissolved in 200 mL deionized water, mixed with the previous solution at 80 °C for 5 h, and heated to 90 °C for 19 h with condensation. The mixture was aged without heating for 12 h, filtered and washed with deionized water, and dried overnight in a vacuum at 45 °C. The dried material was granulated using ALG as the binder in a reaction path, as shown in Figure 19, according to the procedure reported by Luo et al. [24].

The non-granulated and granulated Li/Al–LDH LISs were characterized by FTIR spectroscopy, XRD, SEM, TEM, XPS, and nitrogen adsorption studies. The adsorption properties of the powdered and granulated LISs were evaluated using LiCl as the sorbate, 0.5 g of the adsorbent, concentration range of 20–500 mg/L, shaker speed of 200 rpm, pH 4–10, and temperature in the range of 25–45 °C. The results show that Li adsorption increased with an increase in temperature and Li concentration to a maximum of 15.52 mg/g at 45 °C, in agreement with the study reported by Luo et al. [24]. The presence of competing ions had no significant effects on the adsorption properties of the granulated LISs. Reusability studies showed that the granulated LIS maintained 92.2% of the initial adsorption capacity after six adsorption–desorption cycles. The adsorption properties of the granulated LISs were further evaluated using East Taigener Salt Lake brine with a Li concentration of 1400 mg/L. The results show that adsorption equilibrium was achieved within 240 min, where the adsorption process was best described by the pseudo-second-order adsorption kinetic model. The maximum Li adsorption capacity was noted as 14.5 mg/g. The results further affirmed the high selectivity of the granulated LISs for Li-ions, as evidenced by the minimal difference in the concentration of competing ions in the brine before and after the adsorption process. Furthermore, XRD results before and after 10 adsorption–desorption cycles supported the mechanical and chemical stability of the granulated LIS adsorbents. The chemical and mechanical stability of the granules prepared by Luo et al. [24] and Li et al. [72] are very similar as expected because of the very similar synthetic strategies employed by the authors, where crosslinking was performed before granulation. In particular, the granules prepared by both authors were from Al-based precursors and displayed little or no attenuation of adsorption properties after 10 adsorption–desorption cycles. The results of these studies further affirm the role of crosslinking before granulation on the chemical and mechanical stability of granulated adsorbents, as previously highlighted by the studies of Xie et al. [52] and Ding et al. [49].

**Figure 20:**
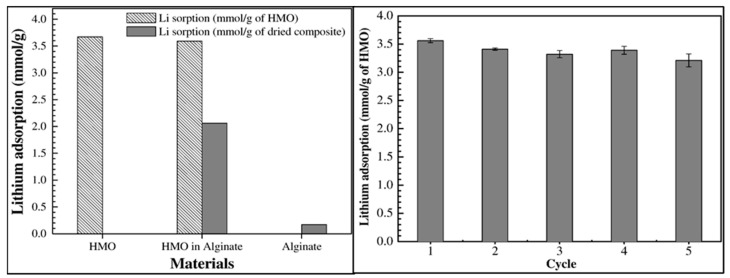
Comparison of the adsorption capacity of Li^+^ to different materials. Initial Li^+^ concentration 4.23 mM, volume 20 mL, the mass of HMO 20 mg, the mass of wet HMO–Al beads 0.56 g (corresponding to 20 mg HMO), pH 8.5, and time 24 h. Change in the Li^+^ adsorption capacity to HMO–ALG beads with reuse time. For adsorption: Initial Li^+^ concentration 4.23 mM in 0.1 M CaCl2, volume 20 mL, mass of wet HMO–ALG beads 0.56 g (corresponding to 20 mg HMO), pH 8.5, and time 24 h. For desorption: 40 mL mixture of 0.1 M HCl and 0.1 M CaCl2 for 12 h (reproduced from [73] with permission).

Koilraj et al. [73] reported the encapsulation of powdery spinel-type LISs derived from biogenic manganese oxide (LMO) in ALG beads. The biogenic LISs were granulated using ALG as the binder as follows: Following acid treatment of the biogenic LMO for 12 h to obtain the biogenic HMO, approximately 0.4 g of the HMO was suspended in 20 mL of deionized water and continuously stirred for 1 h. 0.35 g of sodium ALG (1.5 *w*/*v*%) was then added to the mixture at 60 °C under vigorous stirring for 1 h to obtain a homogenous LMO–sodium ALG suspension. Bead formation was achieved by slow-dropping the suspension into 200 mL 0.2 M CaCl_2_ solution using a disposable syringe with an inner diameter of 0.8 mm. The resulting beads were aged for 12 h, washed three times with decarbonized water to remove unreacted chemicals, and stored in deionized water prior to use. Blank alginate (ALG) beads without the HMO LISs were also prepared. Characterization of the powdery and granulated adsorbent materials was achieved by XRD, SEM and TEM studies. Batch adsorption studies with a solution containing 4.23 mM Li ions and the granulated biogenic HMO–ALG LIS, powdery HMO, and ALG beads at a solid-to-liquid ratio of 1.0 g/L were performed, and results are shown in Figure 20. The results show that HMO powder had a maximum adsorption capacity of 3.67 mmol/g, 0.17 mmol/g for ALG beads, and 3.59 mmol/g for granulated HMO–ALG LIS.. The results show that the adsorption of Li ions by HMO–ALG LISs mainly occurs in the HMO domain of the granulated LISs, whereas encapsulation of HMO in the sodium ALG matrix did not negatively impact the adsorption properties of HMO LISs. The change in adsorption properties of the granulated LISs due to multiple usage was evaluated, and the results reveal that there was a slight decline in the adsorption capacity with an increase in the number of adsorption–desorption cycles (cf. Figure 20). The authors attributed the slight decline to Mn loss through dissolution due to acid treatment during desorption. Furthermore, the granulated LISs maintained more than 90% of their adsorption capacity in the fifth cycle, consistent with the results of non-granulated LISs reported in a previous study [102]. The synthetic strategy reported by Koilraj et al. [73] is slightly different from that of Luo et al. [24] and Li et al. [72], where the later authors used Al-based LIS precursors relative to Mn-based precursor by Koilraj et al. [73].

In another study, a novel strategy was employed to separate Li ions from concentrated seawater using a synergistic effect of Al^3+^- crosslinked ALG and hydrogen manganese oxide (HMO) [74]. The crosslinked Al^3+^ in the ALG network structure generated a strong repulsive force to debar cations from competition with Li ions for adsorptive binding sites in the composite adsorbent. Preparation of the LIS–ALG composites was performed as follows: 0.5 g of LIS (LMO) was added to 400 mL of 2.5 wt% aqueous ALG solution. The mixture was vigorously mixed with the aid of a sonicator. Granulation was carried out by dropping the mixture into a 5 wt% AlCl_3_ solution using a peristaltic pump. The prepared granulated LIS was washed with deionized water until the conductivity of the wastewater was similar to that of deionized water. The granulated LISs were dried in a humidity oven operating at room temperature and relative humidity in the range of 65–70%. De-lithiation was carried out by treatment of 0.4 g of the granulated LIS with 400 mL of 1.0 M HCl. Following de-lithiation, the granulated HMO/ALG(Al) LISs were washed with deionized water and dried at room temperature. Another sample (HMO/ALG(Zr)) was prepared through the same procedure, except changing the crosslinking agent to ZrCl_4_. A control sample, ALG(Al), was prepared according to the same procedure but without the addition of LMO. The structural and morphological properties of the synthesized materials were elucidated through XRD, TEM, and SEM studies.

**Figure 21:**
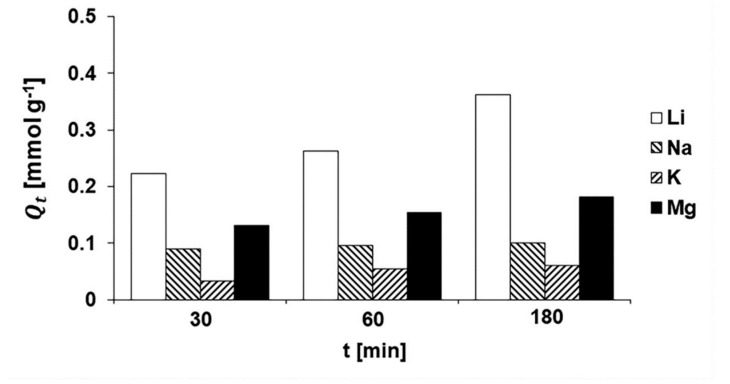
Comparisons of adsorption capacities of HMO/ALG(Al) for Li^+^, Na^+^, K^+^, and Mg^2+^ cations from multi-ion solutions containing 750 mg L^−1^ of each salt cation according to incubation time. (Reproduced from [74] with permission).

The effect of competing ions on the adsorption properties of HMO/ALG(Al) was evaluated by measuring the concentrations of Li^+^, Na^+^, K^+^, and Mg^2+^ in a multi-ion solution matrix where the initial concentration of each ion was 750 mg L^−1^. The results show that the adsorption capacities for Li ions at 0.5, 1, and 3 h were 0.223, 0.263, and 0.363 mmol/g, respectively, where the adsorption capacity increased more than 1.6 times at the end of a 3 h incubation period. On the other hand, the adsorption capacities for Na^+^, K^+^, and Mg^2+^ increased 1.1, 1.8, and 1.4 times for various incubation times (30–180 min) (cf. Figure 21). The results suggest that the granulated HMO/ALG(Al) can selectively adsorb Li ions in the presence of other cations, though with a lower affinity for Li ions relative to other recently developed LISs [103]. The choice of various LIS granulation techniques suggests that numerous LIS materials with diverse properties and applications may be synthesized.

The adsorption property of the granulated HMO/ALG(Al) sieves was also evaluated using concentrated seawater with a Li concentration of 4 mg/L and more than 100 g/L of competing ions. The results show that the granulated HMO/ALG(Al) LISs display a high affinity for Li ions with the selectivity separation factors of 36.36 for Li vs. Na (*α_Na_^Li^*) and 40.08 for Li vs. K (*α_K_^Li^*), along with a negligible adsorption capacity for Mg ions, respectively. This result demonstrates high selectivity for Li ions from a complex matrix. However, during the second cycle, the Li-ion selectivity largely decreased because a large amount of Na^+^, K^+^, and Mg^2+^ ions were captured. The authors attributed the drop in selectivity of the granulated LIS to a compromise on structural integrity due to extensive swelling that may result in the extraction of HMO particles from the matrix. To affirm the claim of compromise on the structural integrity of HMO/ALG(Al), the authors evaluated the adsorption properties of another granulated LIS crosslinked with ZrCl_4_. The results show that the adsorption capacity for Li ions increased while the competing ions were rejected, where the selectivity separation factors *α_Na_^Li^* and *α_K_^Li^* for HMO/ALG(Zr) increased from 3.71 and 5.78 at the first cycle to 36.9 and 70.0 during the third cycle, respectively. The authors claimed that the increased selectivity for Li ions by HMO/ALG(Zr) may be attributed to the reorganization of the Zr^4+^–ALG network or to the continued de-lithiation through repetitive acid exposure, which is completely different from the behavior of HMO/ALG(Al). In addition, the Zr^4+^–crosslinked ALG network structure enhances structural stability and reusability due to ionic crosslinking between Zr^4+^ ions and two, three, or even four carboxyl groups of ALG [53]. Thus, this structural coordination with many carboxyl groups induces very low swelling compared with the Al^3+^–crosslinked ALG composites. The results reported by Park et al. [74] further highlight the role of crosslinkers on the chemical and mechanical stability as well as selectivity for Li ions by granulated adsorbents.

#### 3.2.4. Metal-Based LISs Granulated with Agar

**Figure 22:**
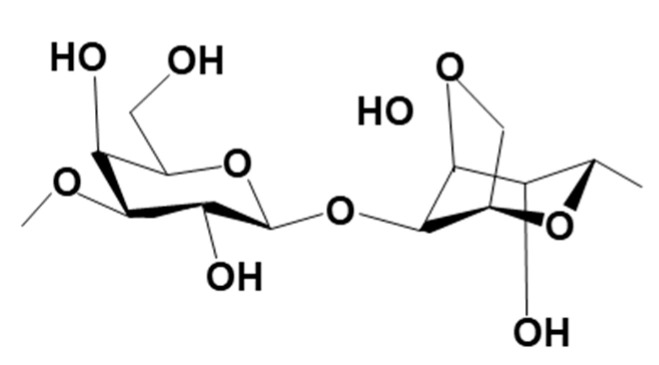
Molecular structure of Agar.

Agar (AG) is a biopolymer that belongs to the polysaccharide family and is extracted from specific species of marine red algae from the Rhodophyceae class [104,105]. The two main commercial sources of this biopolymer are *Gelidium* sp. and *Gracilaria* sp. AG forms a supporting structure in the cellular walls of the seaweeds [104,106,107]. Its chemical structure consists of a mixture of agaropectin (non-gelling fraction) and agarose (gelling fraction) [106,108]. Agarose is a linear polysaccharide consisting of repetitive units of D-galactose and 3-6-anhydro-L-galactose, linked by alternating α-(1 → 3) and β-(1 → 4) glycosidic bonds (cf. Figure 22). On the other hand, agaropectin is slightly branched and sulfated. Interest in the use of AG as a binder for the granulation of LISs relates to its gel-forming properties in hot water, where the gel-forming ability is a result of its ability to form hydrogen bonds between agarose molecules [104,106,109]. This section of the review will highlight recent progress in the application of AG as binders for the granulation of metal-based LISs.

Various studies have reported on the granulation of LISs using AG as the binding agent. For example, Han et al. [76] have prepared AG-based mm-sized spherical ion-sieve foams (SIFs) with hierarchical pore structures from LMO for the recovery of Li ions from seawater. The granulated LISs were prepared via a mechanical foaming method, drip-in-oil, and AG gelation processes [110]. According to the authors, a 60 wt.% slurry of LMO was prepared using deionized water, and the temperature of the slurry was maintained at 60 °C. Foaming was achieved via the addition of 4 mL/L of sodium lauryl sulfate to the slurry, followed by the addition of AG solution of different concentrations (2, 3, 4, or 6%). The slurry was granulated by dripping into liquid paraffin solution (height = 50 cm and temperature = 5 °C) at a flow rate of 10 cm^3^ min^−1^ using a syringe with an inner diameter of 2.4 mm. During foaming and dripping, the slurry temperature was maintained at 60 °C. The resulting granulated and gelled LIS spheres were collected from the bottom of the liquid paraffin column, dried at room temperature for a week, and then washed in water/ethanol at 5 °C for 1 h to remove any oil and surfactant from the samples. De-lithiation was achieved by treating the granules with 0.5 M HCl at 25 °C for 5 days, followed by drying at room temperature for 24 h. The LMO obtained after acid treatment was designated as HMO, while the granules were designated as (SIFs)-*x*, respectively, where *x* indicates the mass percentage of AG in the granules. The starting materials and the granules were characterized via XRD, FE-SEM, mercury porosimetry, pycnometry, and nitrogen adsorption–desorption studies.

**Figure 23:**
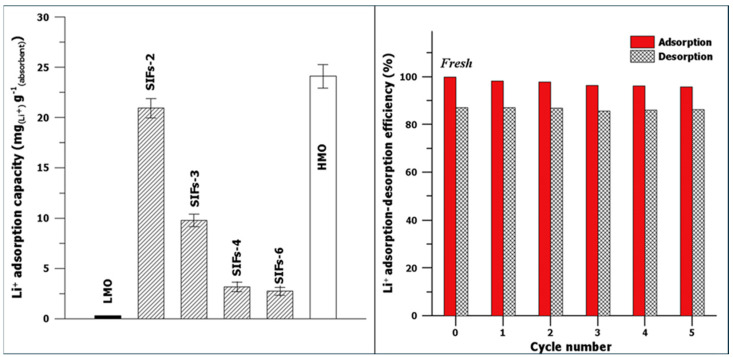
Lithium adsorption capacities for LMO, HMO, and SIFs with different agar contents. The tests for Li^+^ adsorption capacity were carried out in 0.1 M LiOH (pH 13) for 24 h at 25 °C. Li^+^ adsorption–desorption efficiency of SIFs-2 in natural seawater as a function of the cycle number. The red and grid bars represent the adsorption and desorption efficiencies, respectively (reproduced from [76] with permission).

Evaluation of the adsorption properties of LMO, HMO, and the SIFs was performed using 0.1 M LiOH at pH 13. The results of adsorption studies with LiOH show that uptake capacity was negligible for LMO, 24.1 mg/g for HMO, 20.9 mg/g for SIFs-2, and 2.7 mg/g for SIFs-6. The results indicate that the adsorption capacity of HMO was greater than that of SIFs-2, which exhibited the best adsorption capacity among the SIFs with different AG contents. Due to the hierarchical pore structure and highest uptake capacity, SIFs-2 granules and HMO were used for the adsorption experiments with seawater with a Li concentration of 0.17 mg/L at pH 8. The results reveal that HMO and SIFs-2 had adsorption capacities of 16.7 mg/g and 3.4 mg/g, respectively. Furthermore, the reusability of SIFs-2 granules was tested with seawater, where the results indicate a minor loss (4.2%) in adsorption capacity, while the desorption efficiency was maintained at approximately 86% over five adsorption–desorption cycles, as shown in Figure 23.

**Figure 24:**
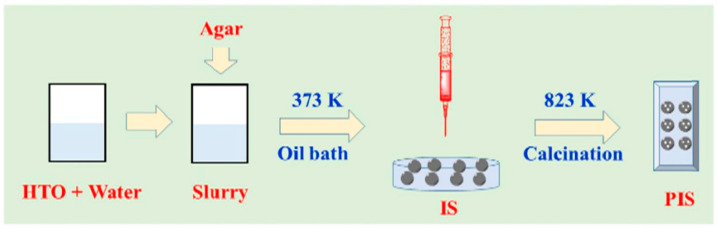
Schematic diagram of the synthesis of titanium-based ion sieves (ISs) and porous ion sieves (PISs) (reproduced from [77] with permission).

The adsorption properties of Ti-based LISs identified as ion sieves (ISs) and porous ions sieves (PISs) with enhanced post-separation ability and high-performance Li recovery from geothermal water were reported by Chen et al. [77]. The synthetic procedure for ISs and PISs is schematically shown in Figure 24 and described as follows: Li_2_TiO_3_ (LTO) precursor was de-lithiated using 0.25 M HCl and a mass/volume ratio of 1 g/L at 333 K for 12 h to obtain the corresponding HTO powder. Following de-lithiation, the HTO powder and AG were mixed at variable mass ratios (1:1, 2:1, 3:1, 4:1, and 5:1) using deionized water and heated to 373 K with stirring until the AG became molten. The resulting molten slurry was granulated by dropping it into cold simethicone via an injector. The granules were left in the granulation solution for 24 h, cleaned using petroleum ether, and dried to obtain the granulated LISs (IS-1 to IS-5). The corresponding PIS was obtained after calcination of IS in air at 823 K for 1 h (cf. Figure 24).

**Figure 25:**
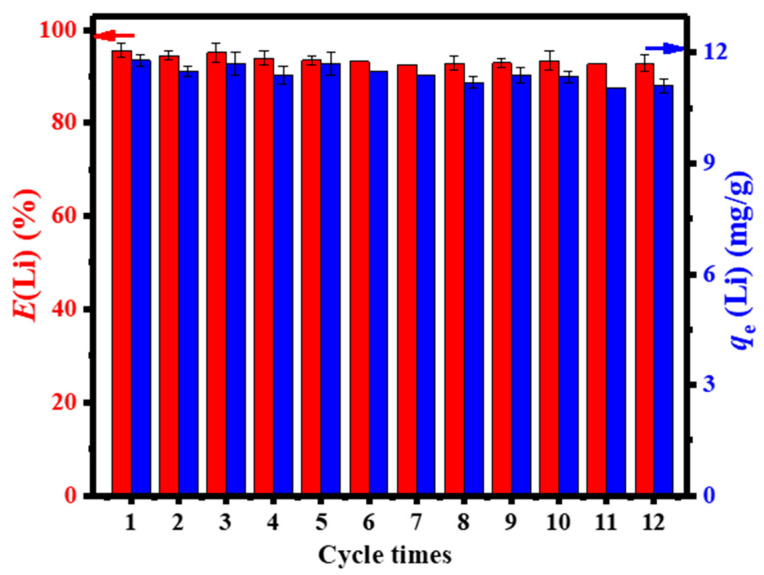
The reusability of PIS-4 with 12-times adsorption–desorption cycles (reproduced from [77] with permission).

Structural and morphological properties of the granulated LISs were elucidated via XRD, TGA, TG–DSC, SEM, TEM, nitrogen adsorption, and XPS studies. Adsorption properties of the granulated LISs (ISs and PISs) were evaluated at pH 12 and 333 K. The Li adsorption capacity of IS and PIS increased with increasing concentration of HTO and achieved a maximum at HTO: AG ratio of 4:1 in both IS and PIS, where the adsorption performance of PIS was better than that of IS because of its mesoporous structure. Due to the superior adsorption capacity of PIS-4, it was used for further studies. Further experiments revealed that the adsorption process was favored at alkaline pH conditions and uptake increased with increasing temperature and Li-ion concentration in solution. The adsorption equilibrium was achieved in 4 h. Based on the Langmuir adsorption model, the maximum adsorption capacity of PSI-4 was 34.23 mg/g. The granulated LIS (PIS-4) was further evaluated for its ability to recover Li ions from geothermal water containing competitive ions (Na^+^, K^+^, and Ca^2+^), where the initial concentrations of Li^+^, Na^+^, K^+^, and Ca^2+^ in the geothermal water were 25.8, 682.1, 137.9, and 211.1 mg/L, respectively at pH 8.80. The pH of the geothermal water was adjusted to 12 using NaOH solution. The Li ion removal efficiency of PIS-4 was 95.3%, while the separation factors for competing ions were 1162.3, 273.7, and 328.5 for Na^+^, K^+^, and Ca^2+^, respectively, in agreement with the ion sieving effect of PIS-4. To further evaluate the effects of competing ions like Mg^2+^, a binary solution containing Li and Mg ions was prepared, where the concentrations were fixed at 25 and 50 mg/L for Li^+^ and 100, and 200 mg/L for Mg^2+^, respectively. The results obtained show that increasing the concentration of Mg^2+^ in solution had negligible effects on the adsorption capacity of PIS-4. The reusability of the granulated PIS-4 was evaluated using variable concentrations of HCl solution. The results show that the desorption efficiency of Li ions gradually increased with increasing HCl concentration upto 0.25 mol/L, where the increase was negligible, but the dissolution of Ti increased significantly. Furthermore, it was noted that PIS-4 was more chemically stable than HTO. HTO had a 4.2% mass loss due to Ti dissolution in 0.5 M HCl relative to 1.2% for PIS-4. Due to Ti dissolution at high HCl concentrations, 0.25 M HCl was chosen as the desorption solution. The authors reported that the adsorption and desorption efficiencies of PIS-4 did not display a significant change after 12 adsorption–desorption cycles, as evidenced in Figure 25.

## 4. Comparison of the Sorption Properties of Granulated LISs

In this section, we report the adsorption properties of various granulated LISs to compare the efficiency of the different types of binding and crosslinker agents. Table 1 summarizes the uptake capacities for a variety of granulated LISs that were prepared from various biopolymer and crosslinker agents and evaluated in different brine adsorbate solutions. The crosslinker molecules used include sulfuric acid, ECH, EDGE, and GLY, while spiked seawater, concentrated seawater, geothermal water, and LiCl were used as adsorbates. The results show that the LISs granulated with CTS as the binder displayed adsorption capacities in the range of 5 to 54.7 mg/g, where the highest adsorption capacity was displayed by the granules crosslinked with sulfuric acid. The CTS-based granules crosslinked with ECH exhibited the most efficient adsorption–desorption performance at six cycles, where the performance may be related to the two-step crosslinking strategy. On the other hand, the LISs granulated with various forms of CLS revealed uptake capacities in the range of 12 to 54.2 mg/g, with the highest adsorption capacity and reusability (20 adsorption–desorption cycles) displayed by the granule crosslinked with N,N’-methylenebis (acrylamide) (MBA). In the case of ALG based granulated LISs, the adsorption capacity was in the range of 2.52 to 24.9 mg/g, where CaCl_2_ was the most common crosslinker used for the granulation process. As for the AG-based granulated LISs, the adsorption capacities were estimated between 3.40 to 12.3 mg/g, respectively. One of the challenges faced in the direct comparison of adsorption capacities is the use of variable adsorption conditions such as adsorbate concentration and type, pH, as well as the solid (adsorbent) to liquid (adsorbate) ratios during the studies. This challenge limits the direct comparison of the granulated adsorbents and identification of the most suitable binder for the LISs presented in this review.

## 5. Prospects and Challenges

One of the challenges in the use of LISs for direct Li extraction is the phase separation of powdered-form LISs following extraction and recovery of Li ions from aqueous media. Several techniques have been employed towards the mitigation of this challenge. One of which is granulation, where the use of synthetic organic polymers, inorganic materials and biopolymers has been reviewed. This study has revealed chitosan, cellulose, alginate, and agar as the most widely used biopolymers for the granulation process, where a majority of the literature reports are based on the use of chitosan as the binder. These reports, which cover the last 10 years, have highlighted the prospects of granulation in improving the performance of LISs through modifying the textural/surface and mechanical properties of the granulated bio-composite materials. It has also been demonstrated that the ratio of binder-to-Li ion sieves plays an important role in determining the adsorption properties, and thus the performance, of the granulated composite materials. Similarly, the acidic pH profile of the eluent solution determines the safe and efficient regeneration of the used LISs for multicycle applications. While these are promising features for granulated LIS bio-composites, there are still gray areas and knowledge gaps that exist in this research area. These can be summarized as follows: (1) current research efforts have not been able to identify the most suitable material to use for the granulation process; (2) there is poor understanding of the most appropriate granulation strategy that will produce composite materials with good mechanical/structural, adsorption capacity, and regeneration properties with multiple cycling performance; (3) there are no reports on the perspective or challenges on the transition of findings from the laboratory to pilot or industrial-scale application. Reports available in the open literature show that different crosslinkers, binders, and solvents have been employed, with no report confidently identifying a strategy or biopolymer granulation agent as the most suitable. Similarly, there are no reports that comprehensively evaluate the effects of granulation on metal loss as well as the effects of variable levels of crosslinking on the adsorption properties of the granulated composite materials. These areas require further research to provide more understanding of the structure–function properties of the biopolymer–LIS composites and their application in the lithium extraction industry. The current study lays the foundation and provides the scope for future prospects and developments.

## 6. Conclusions

This study has highlighted recent progress in the use of various biopolymers, namely chitosan (CTS), cellulose (CLS), alginate (ALG), and agar (AG), as binders for the granulation of LISs. Granulation is the most viable method for producing mechanically stable composite LIS microspheres with improved adsorption–desorption performance from micro/nano-based powdered sieves. Various granulation processes have been reported for lithium manganese oxide (LMO)– and lithium titanium oxide (LTO)-based sieves employing synthetic binders and biopolymers (i.e., CTS, CLS, ALG, and AG) as the binding agents to achieve materials with improved adsorption and mechanical properties. Generally, biopolymers have diverse functional groups (e.g., OH, NH, and C=O) that can be involved in H-bonding and crosslinking reactions to form three-dimensional structures with a multitude of active binding sites for Li templation. They are also highly abundant and less costly relative to synthetic binders, thus supporting favorable economics for Li production at an industrial scale. The structural and performance properties of the biopolymer-granulated LISs were characterized using spectroscopic (e.g., FTIR), imaging (SEM), X-ray diffraction, batch adsorption studies, and adsorption-desorption tests using acids as eluents. While variable adsorbates (e.g., brine, LiCl, LiOH, and seawater, etc.) and adsorption conditions (e.g., temperature, pH, and solid-to-liquid ratio, etc.) were used for the studies, It is concluded that modification of the adsorption performance for the various biopolymer-granulated LISs varies in accordance with the following factors: (1) the presence of abundant active binding sites in the LISs structure, (2) the surface and textural properties of the LISs material, and (3) the nature of the adsorbate matrix. Similarly, the recyclability (adsorption–desorption) performance is accounted for by (1) the nature of the coordination interactions between the Li ion and the adsorption site, (2) the structural integrity/morphology of the LISs granules, and (3) the mechanical/chemical stability of the three-dimensional structure of the granulated LISs. The mechanical/chemical stability of the LISs can be further enhanced through crosslinking of the sieves pre- and/or post-granulation. Several crosslinker agents, such as sulfate anions, GLY, EDGE, CaCl_2_, FeCl_3_, AlCl_3_, and ZrCl_4_, have been employed in this study with various performance outcomes. The choice of the crosslinker is essential to the desired LISs properties in terms of the adsorption–desorption performance and their selectivity for Li ions in a matrix containing competing ions (e.g., Na, K, Mg, and Ca). However, it is generally concluded from this review study that crosslinking prior to granulation leads to LIS materials with greater stability. In particular, a multi-step crosslinking strategy employed prior to and post-granulation can afford composite LIS materials with overall better adsorption properties and structural integrity due to the introduction of multiple active binding sites for Li ions, along with improved stability of the heavily crosslinked composite granules. Finally, the review reveals that there are no studies on the use of CTS as a binder for the granulation of LTO and Li/Al–LDH based LISs materials, use of ALG for the granulation of LTO-based LISs as well as the use of AG for Li/Al–LDH based LISs materials. This, therefore, calls for more studies on the use of these abundant and environmentally friendly biomaterials as binders for the granulation of metal-based Li ion sieves to unlock its potential for the Li mining industry.

## Figures and Tables

**Table 1:** Uptake Capacities of Selected Granulated LIS.

S/N	Binder	Crosslinker	Sorbate	Uptake Capacity (mg/g)	Reference
1	Chitosan	Sulfuric acid	Spiked seawater	10.0	Hong et al. [47]
2	Chitosan	Sulfuric acid	Spiked seawater	54.7	Ryu et al. [48]
3	Chitosan	ECH and EDGE	Geothermal water	11.4	Ding et al. [49]
4	Chitosan	ECH	LiCl	5.0	Yüksel Özşen and Kahvecioğlu [50]
5	Chitosan	EDGE, ECH and glutaraldehyde	Geothermal water	8.98	Wang et al. [51]
6	Chitosan	ECH	LiCl	4.94	Recepoğlu et al. [78]
7	Cellulose	NA	LiCl	28.6	Qian et al. [69]
8	Carboxymethyl cellulose	FeCl_3_, EGDE, and ME	Geothermal water	12.0	Xie et al. [52]
9	Bacterial cellulose	NA	LiCl	35.5	Zhang et al. [70]
10	Hydroxyethyl cellulose	MBA	LiCl	54.2	Liu et al. [71]
11	Sodium alginate	CaCl_2_	Salt Lake brine	9.16	Luo et al. [24]
12	Sodium alginate	CaCl_2_	Salt Lake brine	14.5	Li et al. [72]
13	Sodium alginate	CaCl_2_	LiCl	24.9	Koilraj et al. [73]
14	Sodium alginate	AlCl_3_/ZrCl_4_	Concentrated seawater	2.52	Park et al. [74]
15	Agar	NA	Seawater	3.40	Han et al. [76]
16	Agar	NA	Geothermal water	12.3	Chen et al. [77]
